# L-Arginine and L-Citrulline Supplementation Have Different Programming Effect on Regulatory T-Cells Function of Infantile Rats

**DOI:** 10.3389/fimmu.2018.02911

**Published:** 2018-12-10

**Authors:** Yi-Chen Lee, Yu-Tsun Su, Ta-Yu Liu, Chih-Min Tsai, Chih-Hao Chang, Hong-Ren Yu

**Affiliations:** ^1^Department of Pediatrics, Chang Gung Memorial Hospital–Kaohsiung Medical Center, and Graduate Institute of Clinical Medical Sciences, Chang Gung University College of Medicine, Taoyuan, Taiwan; ^2^Department of Pediatrics, E-Da Hospital/I-Shou University, Kaohsiung, Taiwan; ^3^Department of Respiratory Therapy, Chang Gung Memorial Hospital–Kaohsiung Medical Center, Kaohsiung, Taiwan

**Keywords:** L-arginine, L-citrulline, Treg, infant, rat

## Abstract

Arginine is a semiessential amino acid in healthy adult human, but is essential for preterm, newborn or critically ill patients. Arginine can be supplied from our diet or *de novo* synthesis from citrulline. In conditions of sepsis or endotoxemia, arginine may be deficient and be accompanied with altered immune response. L-arginine supplementation can ameliorate dysregulated immune condition and improve prognosis. Many studies had tried L-arginine or L-citrulline supplementation to examine the effect on immune response in the adult population. Few had studied on the young children. In this study, we determined the effect of L-arginine and L-citrulline supplementation on the immune response of infantile rats. Male infantile rats received normal saline, L-arginine (200 mg/kg/day) or L-citrulline (200 mg/kg/day) intraperitoneally over postnatal day 8 to day 14. The infantile rats were then sacrificed. The blood was analyzed while the spleen was indicated for immune analysis after stimulation with concanavalin A (Con A) or lipopolysaccharide (LPS). We found L-arginine supplementation enhanced Th1 immune response by increasing IFN-γ production. Both the L-arginine and L-citrulline therapy can modulate regulatory T-cell (Treg) immune effects by increasing the IL-10 level. Only the L-citrulline group showed a TGF-β1 increase. Both L-arginine and L-citrulline therapy were also noted to decrease SMAD7 expression and enhance SIRT-1 abundance. However, FOXP3 expression was only modulated by L-citrulline treatment. We then concluded that L-arginine and L-citrulline supplementation can modulate the regulatory T-cells function differently for infantile rats.

## Introduction

Arginine is a semi-essential amino acid in human depending on the developmental stage or health status of the individual. Arginine can be derived from dietary intake, from *de novo* synthesis from citrulline and through protein breakdown. It is essential to preterm, newborn or critically ill patients, as preterm/newborns are unable to synthesize arginine, while critical ill patients' arginine are often depleted (1). Arginine has many important roles in several metabolic pathways. For immune system, its deficiency is associated with sepsis and inflammatory conditions (2–5). Arginine deficiency is related to decreased arginine uptake and impaired arginine *de novo* synthesis from citrulline, in combination with an enhanced arginine catabolism by the up-regulation of arginase and the nitric oxide synthase (NOs) in the immune response (6). Arginase and NOs can be induced by cytokines produced by T helper cells. NOs is stimulated by Th1 cytokines such as interferon-gamma (IFN)-γ and interleukin (IL)-1 which play important roles in intracellular defense against microorganisms while arginase is activated by Th2 related cytokines such as IL-4, IL-5, and IL-13 which are responsible for allergic reactions (7, 8). In contrast, the role of regulatory T cells (Treg) is to suppress over-activated effector T cells and play a key role in the regulation of Th1/Th2 immune responses (9–11). Treg secrets regulatory cytokines such as transforming growth factor (TGF)-β and IL-10 (12).

Over the years, many studies have tried to supply arginine with or without other nutrition compounds as a therapeutic strategy to restore the decreased arginine levels in septic and critical ill patients but the results were inconsistent (13). Recent studies examining L-arginine monotherapy in experimental sepsis/endotoxemia of porcine model has shown beneficial effects on the plasma arginine levels without side effects (14). L-arginine supplementation can also enhance immune response, increase protein turnover rate, and elevate NO synthesis (15, 16). However, one study showed a higher mortality rate in severe septic patients with arginine supplementation in the enteral diet (17). With these inconsistent findings, the previous views of L-arginine supplement need to be revisited.

Most of the current studies focus on the effect of L-arginine supplementation for critical ill population in adults. However, study on the neonatal population remains scarce. In our previous study, we had found neonate has lower plasma L-arginine level but more abundant arginase I in polymorphonuclear cells than adult. Exogenous L-arginine could enhance neonate lymphocyte proliferation through an IL-2 independent manner (18). For newborn, whose more susceptible to arginine deficiency than adult, more study about the modulatory effects of L-arginine supplementation are needed.

Supplementation of citrulline as a source of arginine is alternative therapeutic intervention being studied currently. L-citrulline supplementation seems to be more effective than L-arginine supplementation directly for improving arginine level in sepsis. This is supported by studies showing that treatment with L-citrulline in endotoxemic rats resulted in higher plasma arginine concentration than treatment with L-arginine in certain conditions (19, 20). Research on citrulline supplementation for modulation of neonatal immunity is limited.

Arginine availability is essential for a normal immune response, specifically T-cell proliferation and function (21). When arginine is depleted, the result could lead to increased susceptibility to infection (22). Neonatal plasma L-Arginine level was previous shown to be lower than in adults and this can partly explain why newborns more prone to infection (18). Therefore, the importance of arginine and/or citrulline to neonatal immunity cannot be underestimated. More study is needed to explore the influence of exogenous amino acids on neonatal immunity. The aim of this study is to investigate the immune modulatory effects of L-arginine and L-citrulline on infant using a rat model. We found L-arginine and L-citrulline supplementation have different programming effect on regulatory T-cells function of infant rats.

## Materials and Methods

### Animals

This study was conducted in strict accordance with the recommendations outlined in the Guide for the Care and Use of Laboratory Animals of the National Institutes of Health. The protocol was approved by the Institutional Animal Care and Use Committee of the Kaohsiung Chang Gung Memorial Hospital (No.2014102002). Virgin Sprague–Dawley (SD) rats (12–16 weeks old; BioLASCO Taiwan Co., Ltd., Taipei, Taiwan) were housed and maintained in a facility accredited by the Association for Assessment and Accreditation of Laboratory Animal Care International as previously described (23). Virgin SD female rats were mate with male rats for 24 h and then were separated from the male rats and housed individually in a standard plastic home cage. After birth, the male offspring pups were randomly assigned to the L-arginine group (L-Arg), L-citrulline group (L-Cit) or control group (C) at postnatal day 8. For the L-Arg group, L-arginine was administered intraperitoneally (200 mg/kg/day) from postnatal day 8 to postnatal day 14. L-citrulline was administered intraperitoneally (200 mg/kg/day) over postnatal day 8 to day 14 for the L-Cit group. The control group was intraperitoneally injected with normal saline daily over gestational postnatal days 8–14.

### Experimental Procedures and Specimen Collection

All rats of these three groups were sacrificed at postnatal day 15 to assess the immune modulatory effects of indicated amino acid treatment. Body, thymus, and spleen weights were recorded after sacrifice. The spleen tissues were used for further studies, and blood specimens were collected for analysis.

### Peripheral Blood Analysis and Plasma Immunoglobulin Detection

Blood samples were collected in heparin tubes. Total blood cell counts and white blood cell (WBC) differential counts were obtained using Sysmex XT-1800i (Sysmex, Hyogo, Japan) as previous described (24). For lymphocyte subset analysis, leukocytes were stained with the following antibodies: PE-conjugated anti-rat CD3, APC-conjugated anti-rat CD45RA, PE-conjugated anti-rat CD4, and FITC-conjugated anti-rat CD8a. All these antibodies were purchased from BD Biosciences. Data were acquired using a FACSAria I cytometer (Becton Dickinson, Franklin, NJ, USA) and analyzed using Flow Jo software. The levels of plasma immunoglobulins (Ig), including IgG, IgA, and IgM, were analyzed by ELISA (eBioscience, San Diego, CA, USA).

### Splenocyte Culture and Drug Treatment

In this study, we choose Concanavalin A (ConA) to stimulate whole splenocytes as study model. ConA, a plant mitogen, is a selective T-cell stimulant that active T-cells through T-cell receptor (25, 26). We use cytokines production to represent Th immune response. Since immune response is composed of interplay of various cells, whole splenocytes model was selected to imitate the nature condition. Splenocytes were isolated from the whole spleen as previously described (23, 24, 27). In brief, the spleen was washed with phosphate-buffered saline (PBS) and pressed with a syringe plunger through a 30-μm nylon mesh. After erythrocytes were lysed, the remaining splenocytes were washed with PBS again. All spleen cells were counted and 2 × 10^6^ cells/ml were plated in 24-well plates containing RPMI 1640 medium (Gibco) supplemented with 1% non-essential amino acids, 1% pyruvate, 10% heat-inactivated fetal bovine serum, and antibiotics. Cultured splenocytes were stimulated with or without 5 μg/ml of ConA (Sigma Chemical Co., St Louis, Mo.) or 100 ng/ml of lipopolysaccharide (LPS; Sigma). The cell pellets and culture supernatants were collected at the indicated time for further studies.

### 5-Bromo-2′-Deoxyuridine (BrdU) Cell Proliferation Assay

Proliferation of splenocytes was assessed by the BrdU assay (Millipore), as described previously (23, 24). At first, splenocytes (5 × 10^5^ cells/ml) were suspended in a 96-well plate with enriched RPMI-1640 medium. Then, the splenocytes were stimulated with/without 5 μg/ml of Con A or PBS. After 48 h of stimulation, BrdU reagent was added to the proliferating splenocytes and labeled for the following 24 h. Proliferation was measured by the BrdU assay according to the manufacturer's instructions. The results were presented as the ratio of optical density (OD) of Con A stimulation to OD of control for every group.

### Cytokine Analysis

The isolated splenocytes (2 × 10^6^ cells/ml) were plated in 24-well plates containing enriched medium and treated with or without 100 ng/ml of LPS for 24 h or 5 μg/ml of Con A for 24 or 72 h. The cell culture supernatants were collected for cytokines detection related to innate and adaptive immunity using the ELISA assay (R & D Systems, Minneapolis, MN, USA).

### Quantitative Reverse Transcription-Polymerase Chain Reaction (qRT-PCR)

RNA was prepared from splenocytes or spleen tissue of rats. qRT-PCR was performed as previously described. In brief, 5 μg of extracted RNA sample was reversed transcribed with Moloney murine leukemia virus reverse transcriptase. PCR was performed in 20 μl of total reaction volume containing 2 μl of 1:10 diluted cDNA obtained from reverse transcribed RNA, specific primers, 2.5 mM MgCl, and Maxima SYBR Green/Fluorescein qPCR Master Mix (2X) (#K0242, Thermo Scientific, CA, USA). The cycling protocol comprised one cycle of 10 min at 95°C followed by 45 cycles of denaturation for 10 s at 95°C, annealing for 20 s at 55°C, and extension for 20 s at 72°C. The primers were as follows: Mothers against decapentaplegic homolog 7 (SMAD7): 5′- GGA GTC CTT TCC TCT CTC-3′ (sense) and 5′-GGC TCA ATG AGC ATG CTC AC-3′ (antisense); Sirtuin 1 (SIRT-1): 5′- TGT TTC CTG TGG GAT ACC TGA-3′ (sense) and 5′-TGA AGA ATG GTC TTG GGT CTT T-3′ (antisense); forkhead box P3 (FOXP3): 5′-CCC AGG AAA GAC AGC AAC CTT-3′ (sense) and 5′- CTG CTT GGC AGT GCT TGA GAA-3′ (antisense); peptidylprolyl isomerase B (PPIB): 5′-TCA TCG TGG GCT CCG TTG-3′ (sense) and 5′-AGC CAA ATC CTT TCT CTC CTG TAG C-3′ (antisense). Serial dilutions of the standard cDNA were also used for parallel amplifications. The threshold cycles (Ct) were calculated using the LightCycler software (ver. 1.5.0). Standard curves were plotted with Ct-vs.-log cDNA quantities. We employed the comparative Ct method to determine the relative quantification of mRNA expression. The averaged Ct was subtracted from the corresponding averaged PPIB value for each sample, resulting in ΔCt. ΔΔCt was obtained by subtracting the average control ΔCt value from the average experimental ΔCt value. The fold increase was established by calculating 2-ΔΔCt for the experimental vs. control samples.

### Western Blotting

Western blot was performed as previously described (23, 24). Briefly, 50 mg of spleen tissue was homogenized with protein extraction solution (iNtRon biotechnology, Sungnam, Korea) according to the manufacturer's instructions. After determining protein concentrations, 50 μg samples were boiled and subjected to 12% SDS-PAGE for each lane. After transferring and blocking to a polyvinylidene difluoride (PVDF) membrane, the membrane was incubated with anti-SIRT1 (Abcam, Cambridge, MA, USA) at 1:500 for over-night. After washing and incubation for 2 h with peroxidase-labeled secondary antibody diluted 1:1,000 in T-BST, the blot image was obtained using a Bio-Rad Molecular Imager ChemiDocMP and quantified by Image Lab version 5.0 software (Bio-Rad, Richmond, CA, USA).

### Statistics

The data are expressed as mean ± standard error of the mean. The one-way ANOVA test was used when two groups were analyzed. Results with a *p* < 0.05 were considered statistically significant. All statistical tests were performed using SPSS 19.0 for Windows XP (SPSS, Inc., Chicago, IL, USA).

## Results

### Postnatal L-arginine or L-citrulline Supplementation did Not Increase Lymphoid Organ-to-BW Ratio and BW of Infant Rats

At first, we sought to determine whether postnatal L-arginine or L-citrulline supplement will affect the lymphoid organ size. What we found was, after postnatal administration of L-arginine, L-citrulline, or normal saline, the body weight at postnatal day 15 were similar among the three groups (Table 1). There was no difference in the spleen weight or spleen weight-to-BW ratio among the 3 groups.

**Table 1:** Lymphoid organ-to-body weight ratio and body weight of infant rats.

**Group**	**Control**	**L-Arg**	**L-Cit**
BW (g)	28.31 ± 1.21	28.04 ± 0.63	28.92 ± 0.84
Spleen (g)	0.10 ± 0.01	0.11 ± 0.01	0.12 ± 0.01
Spleen/BW (g)	3.34 × 10^−3^ ± 0.00	3.75 × 10^−3^ ± 0.00	4.00 × 10^−3^ ± 0.00^a^

*BW, body weight; L-Arg, L-arginine group; L-Cit, L-citrulline group. N = 9 to 11 for each group*.

a *Control group at p < 0.05*.

### Postnatal L-arginine or L-citrulline Supplementation Modified Leukocyte Subsets of Infant Rats

We then measured the blood cell counts and its differential count. The white blood cell counts, red blood cell counts, and platelet counts at postnatal day 15 showed no significant difference (Table 2). Regarding the white blood cell differential count, the L-Cit group had a lower percentage of neutrophil to lymphocyte ratio than those of the control group and L-Arg group, while both the L-Arg and L-Cit group had higher percentage of monocyte than that of the control group (Table 2). We further performed flow cytometry analysis of leukocytes from offspring using antibodies directed against the indicated cell surface markers (Table 2). Samples were analyzed and compared for CD3, CD4, CD8a, and CD45RA (Ox-33 antibody). The L-Arg and L-Cit groups had higher percentage of CD8a+ cells than control group. In contrast, the percentage of CD4+ cells in the L-Cit group was significantly lower than the other groups (Table 2).

**Table 2:** Blood cell counts and leukocyte subsets of infant rats.

**Group**	**Control**	**L-Arg**	**L-Cit**
WBC (10^3^/μl)	4.48 ± 1.15	2.90 ± 0.26	2.98 ± 0.23
RBC (10^6^/μl)	3.79 ± 0.14	3.93 ± 0.09	3.90 ± 0.11
HGB (g/dl)	9.34 ± 0.45	9.14 ± 0.24	9.16 ± 0.22
HCT (%)	26.80 ± 0.98	28.20 ± 0.69	28.27 ± 0.70
MCV (fL)	70.67 ± 0.68	71.87 ± 0.99	66.17 ± 6.15
MCH (pg)	24.58 ± 0.51	23.29 ± 0.29^a^	23.53 ± 0.21^a^
**WBC DIFFERENTIAL COUNT**			
Neutrophil# (10^3^/μl)	0.77 ± 0.12	0.86 ± 0.13	0.62 ± 0.05
Lymphocyte# (10^3^/μl)	2.52 ± 0.21	2.06 ± 0.13	2.24 ± 0.18
Monocyte# (10^3^/μl)	0.04 ± 0.01	0.08 ± 0.02	0.09 ± 0.01^a^
Eosinophil# (10^3^/μl)	0.02 ± 0.01	0.01 ± 0.00	0.01 ± 0.00
Basophil# (10^3^/μl)	0.01 ± 0.00	0.01 ± 0.01	0.02 ± 0.01
Neutrophil (%)	22.68 ± 2.43	27.52 ± 2.27	21.18 ± 1.46^b^
Lymphocyte (%)	75.36 ± 2.63	69.22 ± 2.44	74.85 ± 1.60
Monocyte (%)	1.06 ± 0.33	2.54 ± 0.55^a^	3.04 ± 0.35^a^
Eosinophil (%)	0.59 ± 0.35	0.21 ± 0.07	0.35 ± 0.10
Basophil (%)	0.31 ± 0.06	0.46 ± 0.32	0.57 ± 0.23
N/L ratio	0.31 ± 0.04	0.41 ± 0.05	0.29 ± 0.03^b^
**LYMPHOCYTE SUBSET**			
CD3^+^ (%)	9.46 ± 0.87	8.17 ± 0.62	7.54 ± 0.59
CD45RA^+^ (%)	8.76 ± 0.75	10.32 ± 1.17	11.13 ± 1.27
CD3^+^CD4^−^CD8a^−^ (%)	11.43 ± 1.38	13.80 ± 2.10	13.62 ± 1.75
CD3^+^CD4^−^CD8a^+^ (%)	18.48 ± 0.50	21.70 ± 0.77^a^	22.78 ± 1.46^a^
CD3^+^CD4^+^CD8a^+^ (%)	11.94 ± 1.19	10.71 ± 0.83	11.79 ± 0.81
CD3^+^CD4^+^CD8a^−^ (%)	58.15 ± 1.40	53.79 ± 2.28	51.81 ± 1.67^a^

*L-Arg, L-arginine group; L-Cit, L-citrulline group; WBC, white blood cells; RBC, red blood cells; HGB, hemoglobulin; HCT, hematocrit; MCV, mean corpuscular volume; MCH, mean corpuscular hemoglobin; N/L ratio, neutrophil to lymphocyte ratio*.

a vs. Control group at p < 0.05;

b *vs. L-Arg group at p < 0.05. N = 9 to 11 for each group*.

### Postnatal L-arginine or L-citrulline Supplementation did Not Change Plasma IgA, IgE, IgG and IgM Levels During Infancy

**Figure 1:**
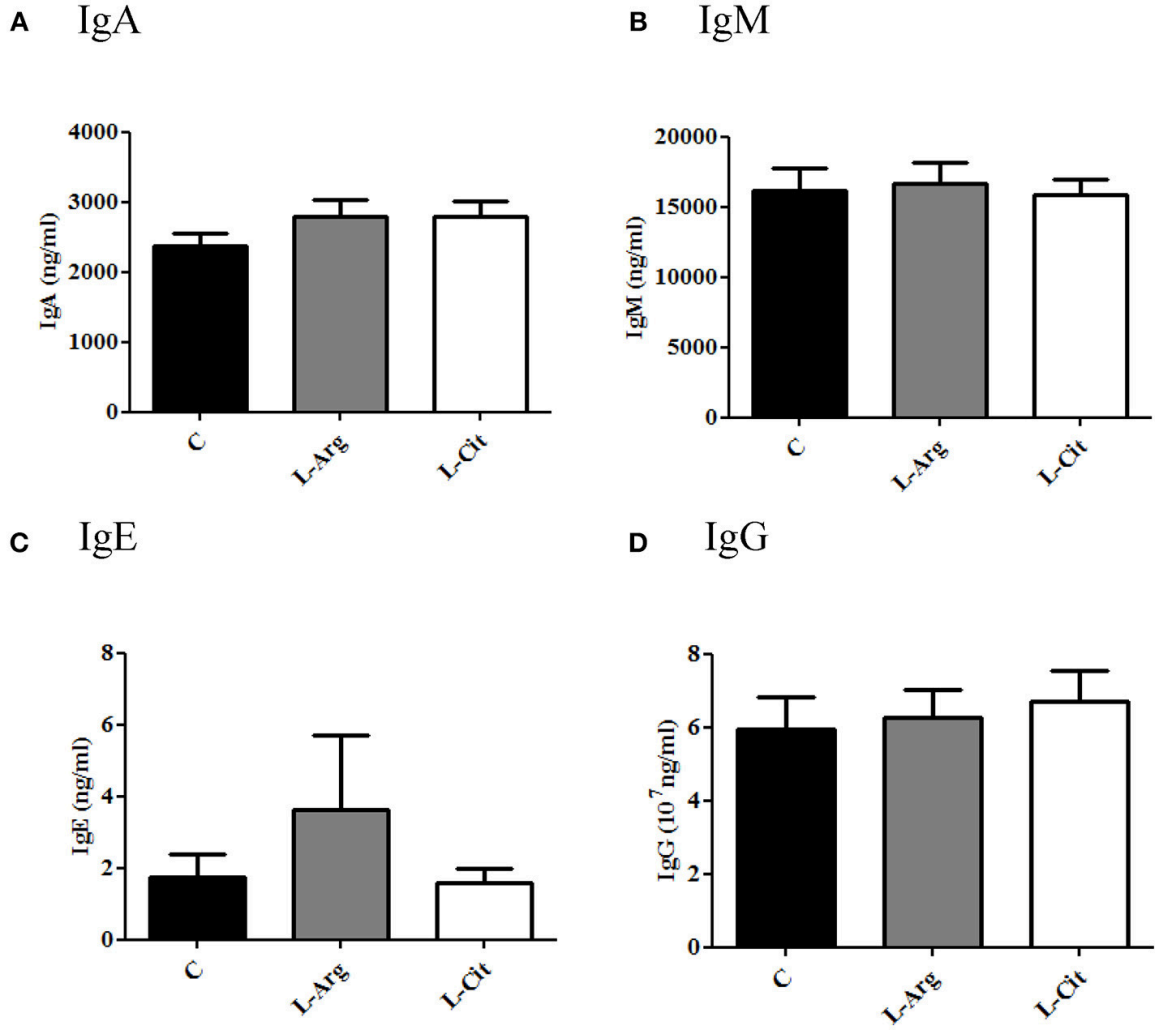
Plasma immunoglobulin (Ig) levels of 15-day-old rats. **(A)** IgA **(B)** IgM **(C)** IgE **(D)** IgG. C, control group; L-Arg, L-arginine group; L-Cit, L-citrulline group. *N* = 9 to 11 for each group.

Total plasma IgA, IgE, IgM, and IgG were measured by ELISA. The IgG level was higher than other immunoglobulins in plasma of infant rats (Figure 1). Overall, the IgA, IgE, IgM and IgG levels showed no significant difference among these three groups.

### Postnatal L-arginine Supplementation During Infancy Enhance Th1 Related Cytokines Production

**Figure 2:**
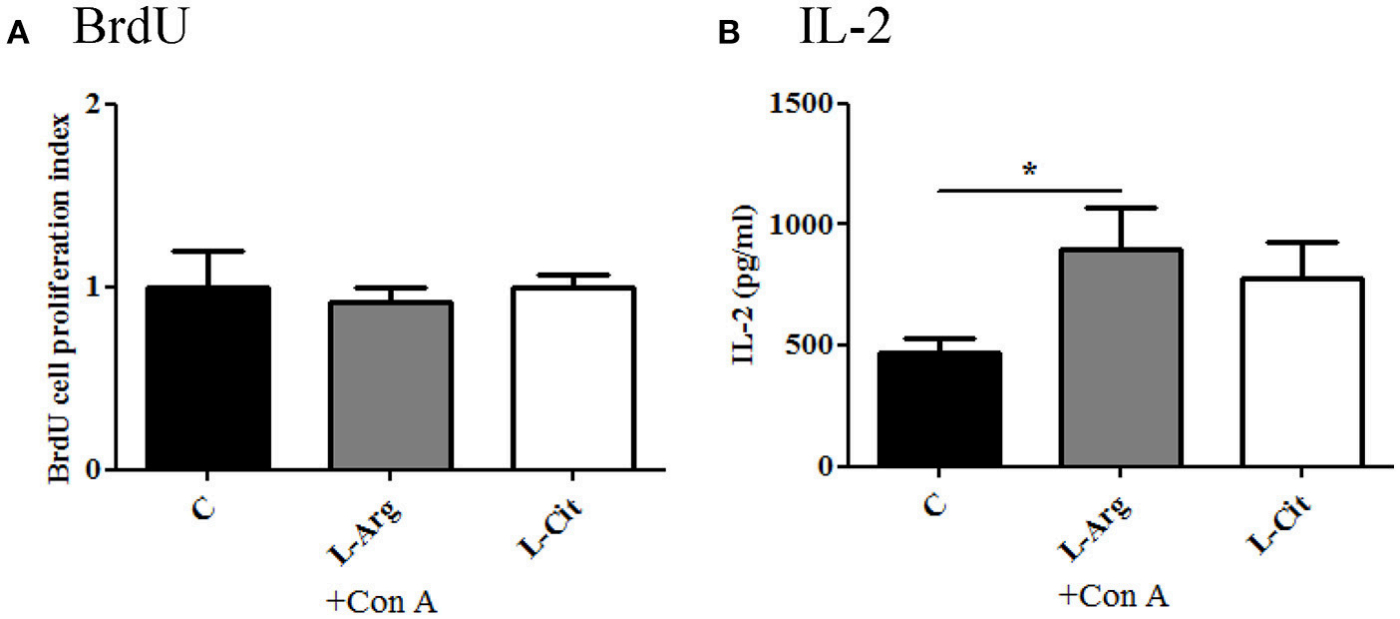
The modulatory effects of L-arginine or L-citrulline supplementation for splenocytes proliferation and IL-2 production on infant rats. **(A)** splenocytes proliferation determined by BrdU array in enriched RPMI-1640 medium. **(B)** IL-2 level in culture supernatants as splenocytes stimulated with 5 μg/ml of Con A for 24 h. C, control group; L-Arg, L-arginine group; L-Cit, L-citrulline group. ^*^p < 0.05. *N* = 9 to 11 for each group.

To test the modulatory effects of L-arginine and L-citrulline on the T cell proliferation and cytokine productions, we first isolated the splenocytes from the whole spleen and maintained in enriched RPMI-1640 medium. To assess the degree of T-cell proliferation, Con A was added to stimulate splenocytes. IL-2 was determined from culture supernatants after stimulation for 24 hrs since IL-2 reach highest level in early stage of proliferation (18, 23). For splenocytes proliferation, BrdU assay was determined after Con A stimulation for 72 hrs. From the BrdU result, we found there is no obvious difference in the optic density regardless of L-arginine or L-citrulline supplement (Figure 2A). However, a discordant result was noted when we tested the IL-2 level and it showed a significant increase only in the L-Arg group (Figure 2B). In this study, we used RPMI 1640 as culture medium, it contains high level of L-arginine (1,150 μM) but without L-citrulline. In previous report, we have shown neonatal T-cell proliferation is in an IL-2 independent manner (18). Thus, the discordant result of IL-2 production and splenocytes proliferation can be explained by the fact that our culture medium contains high level of L-arginine and the unique lymphocyte proliferation response of neonates.

**Figure 3:**
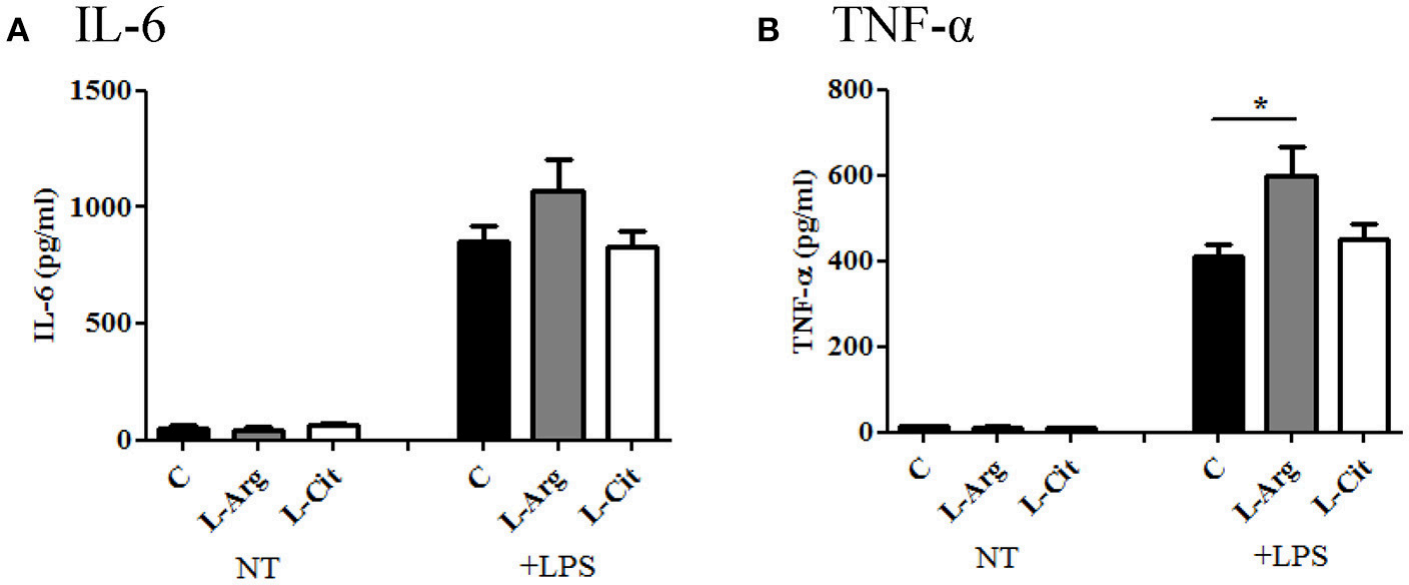
The modulatory effects of L-arginine or L-citrulline supplementation for innate immune cytokine productions on infant rats. **(A)** IL-6 and **(B)** TNF-α productions were determined after splenocytes stimulated in enriched RPMI-1640 medium with LPS for 24 h. C, control group; L-Arg, L-arginine group; L-Cit, L-citrulline group. ^*^p < 0.05. *N* = 9 to 11 for each group.

**Figure 4:**
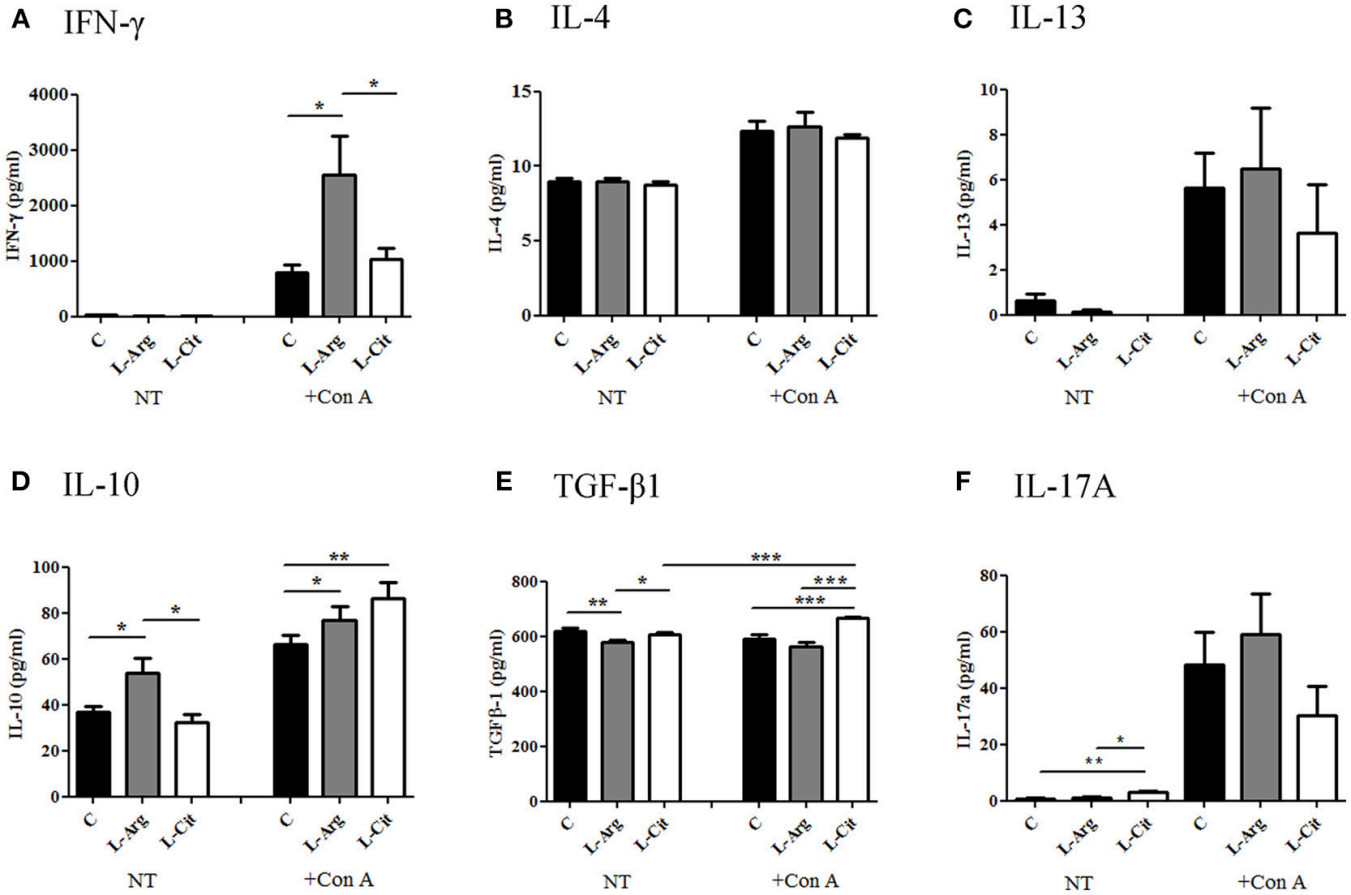
The modulatory effects of L-arginine or L-citrulline supplementation for adaptive immune cytokine productions on infant rats. Th1 related cytokine **(A)** IFN-γ, Th2 related cytokines **(B)** IL-4, **(C)** IL-13, and Treg related cytokines **(D)** IL-10, **(E)** TGF-β were determined as splenocytes cultured with Con A for 72 h in in enriched RPMI-1640 medium. **(F)** IL-17A was represent of Th17 related cytokine. C, control group; L-Arg, L-arginine group; L-Cit, L-citrulline group. ^*^p < 0.05; ^**^p < 0.01; ^***^p < 0.001. *N* = 9 to 11 for each group.

Next, the splenocytes were stimulated with LPS for 24 hrs or Con A for 72 hrs to induce innate and adaptive immune cytokines, respectively. IL-6 and TNF-α were chosen to represent innate immune response. There was no difference in IL-6 production among these three groups (Figure 3A), while TNF-α level was higher in the L-Arg group than the control group (Figure 3B). For Th1 cytokine, IFN-γ was measured and the level was highest in the L-Arg group (Figure 4A). L-citrulline supplement did not enhance IFN-γ production as compared with control group. We assigned IL-4 and IL-13 to be Th2 cytokines. We found that there was no difference in concentration regardless of L-arginine or L-citrulline supplement (Figures 4B,C). The production of IL-17A also did not show a significant difference (Figure 4F).

### Postnatal L-arginine and L-citrulline Supplementation Showed Distinct Treg Immune Modulatory Effects for Infant Rats

When stimulated with Con A, the increase in IL-10 was then seen in both the L-Arg and L-Cit groups when compared to the control group (Figure 4D). L-Cit group showed the highest IL-10 production than other groups. For TGF-β1, when stimulated with Con A, the increase in TGF-β1 level was most profound in the L-Cit group when compared to the control and the L-Arg groups (Figure 4E).

### Postnatal L-arginine and L-citrulline Supplementation Demonstrated Different Modulatory Effects on TGF-β Signaling Cascade

**Figure 5:**
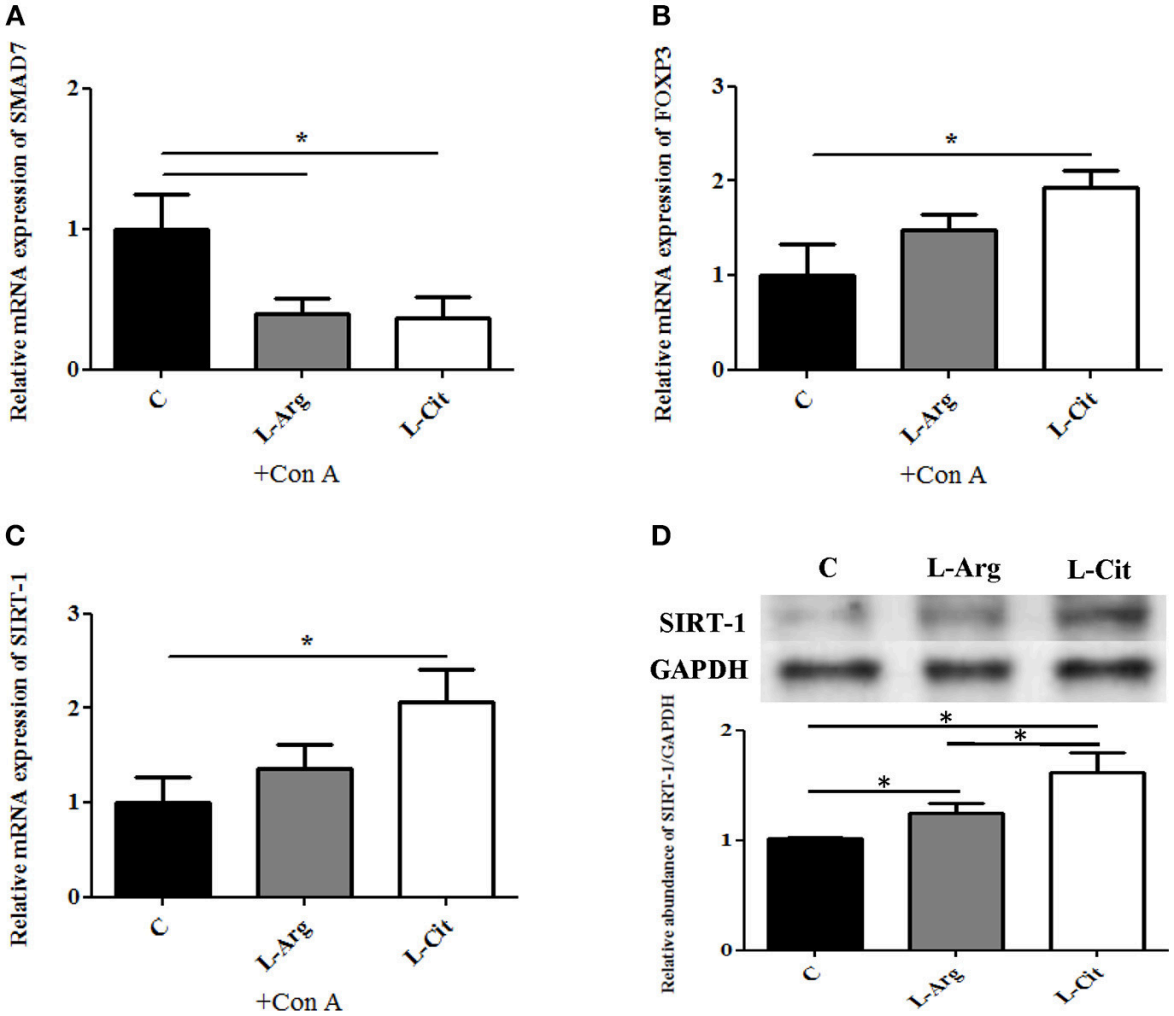
Treg associated regulatory molecules expressions with L-arginine and L-citrulline supplementation. RT-PCR analysis of **(A)** SMAD7 **(B)** FOXP3, **(C)** SIRT-1 mRNA expressions of splenocytes cultured with Con A for 72 h in in enriched RPMI-1640 medium. *N* = 9 to 11 for each group. **(C)** Splenic tissue lysates from 15-day-old infant rats were analyzed by Western blotting with SIRT-1 antibody. C, control group; L-Arg, L-arginine group; L-Cit, L-citrulline group. ^*^p < 0.05. *N* = 9 for each group.

FOXP3 is the key transcription factor for Treg immune. SMAD7 is a transcriptional regulating molecule found mostly in the nucleus, functions as a signal inhibitor for TGF-β receptor (28). To understand the mechanism in which L-arginine and L-citrulline supplementation alter Treg response, qRT-PCR was used to measure the relative expression of SMAD7 and FOXP3 mRNA. As shown in Figure 5A, the expressions of SMAD7 were prominently lowered in both the L-Arg and the L-Cit groups when compared to the control. Furthermore, a higher FOXP3 mRNA expression was seen in the L-Cit group (Figure 5B). We only observed an increasing trend for FOXP3 expression in the L-Arg group but the result not arrive at statistical significance. SIRT-1 is a class III histone deacetylase that resides in the nucleus and can regulate many physiologic functions. SIRT-1 was also reported to regulate immune response through FOX3P modulation recently. Thus, whether SIRT-1 is involved in L-arginine/L-citrulline mediated Treg modulation was studied. L-Cit group revealed a higher SIRT-1 mRNA expression than control group (Figure 5C). Lastly, we measured the relative abundance of SIRT-1 protein in spleen tissue by Western blot. Both L-arginine and L-citrulline supplementation for infant rats enhance the SIRT-1 protein abundance (Figure 5D). L-citrulline treatment conducted to the most abundant SIRT-1 protein as compared with other groups.

## Discussion

Arginine is an amino acid that plays a key role in the immune system. Immune cells such as macrophage and lymphocytes, contains arginase (type I and II) and inducible NO synthase (iNO) that will utilize arginine (29). When arginine is catabolized by arginase, the products are urea, ornithine, polyamines and proline, and when degraded by iNO, the products are a large amount of NO and citrulline (30). In the innate immune system, the NO produced in macrophages and neutrophils is necessary to kill invasive microorganisms (such as viruses, bacteria, and fungi) and tumor cells (21). Markedly increased mononuclear cell arginase activity and decreased plasma arginine/citrulline levels were observed in certain conditions (31). With arginine deficiency, both the innate and adaptive immune responses are impaired and are associated with sepsis and inflammatory conditions such as bacteremia and endotoxemia (2–5). When arginine is deficient, NO production is then limited, thereby increase host susceptibility to invading pathogens (32). In the adaptive immune system, arginine regulates the maturation and proliferation of T and B lymphocytes, the production of cytokines and specific antibodies, the circulating levels of anabolic hormones and the expression of T-cell receptors (CD3ζ) in animals and human (21). Understandably, depletion in arginine can result in the inhibition of T lymphocyte proliferation and IFN-γ production, and the downregulation of CD3ζ, leading to impaired adaptive immune responses in T-cells (33, 34). Arginine depletion also inhibits the proliferation of nature killer cells and their production of IL-12/IL-18 mediated IFN-γ (35). However, with all these studies currently, the majority of them focus on the adult population or the adult animals. Studies that focus on the pathophysiology mechanism and intervention with arginine or citrulline in the pediatric population remain scarce. Recently we found that L-arginine could enhance neonatal Treg related IL-10 production (36). We then tried to mimic the pediatric population with our animal study design which not only explored the effects in which both L-arginine and L-citrulline therapy on innate and the adaptive immune, but also studied the regulatory mechanism of these immune responses. We demonstrated that supplementation of L-arginine and L-citrulline have distinct role in the immune modulation of T cells via cytokines production and regulation in infant rats.

In this study, 200 mg/kg/day of L-arginine and L-citrulline were used for infant rats. These amounts are within the range of doses used in previous literatures (37, 38). Arginine itself is not toxic and its use as a supplement to diets (<2.5% of dry matter) is generally safe for animals (38). Another study suggested short-term use of intravenous arginine at 500 mg of arginine-HCl/kg/day for infants did not result in any harmful effect (39). Based on these findings, with a dietary supplementation with arginine at the doses of 200 mg/kg/day, a 5-kg infant should tolerate supplemental dose of 1 g arginine/d (38).

Citrulline is an amino acid which is a precursor and a metabolite of arginine and its effects in the immune cells are thus partly related to arginine. An impaired conversion of citrulline to arginine by argininosuccinate synthase (ASS) results in immune dysfunction, increased susceptibility to infections and decreased NO production (40, 41). A study by Breuillard et al. showed that citrulline treatment to diabetic fatty rats was able to induce NO production of peritoneal macrophages and modulate macrophage via increasing IL-6 and decreasing TNF-α (42). Similar to the observed decreased arginine concentration in sepsis and endotoxemia, a reduced citrulline production and bioavailability is also noted in sepsis, endotoxemia and inflammatory conditions (20, 43). Several studies had tried to evaluate the supplementation of citrulline in models of sepsis and had found citrulline to be a more productive arginine precursor than arginine (44, 45). Early experimental studies have also suggested its therapeutic potential to restore arginine metabolism in critically ill patients with sepsis (3, 46).

From our study, there was no change in the production of IL-6 while TNF-α showed an increase in production in the L-Arg group. There was no change in the IL-6 and TNF-α production after treatment with L-citrulline. These findings seemed to be different to previous *in vitro* studies of type II DM rats that showed a decrease in TNF-α after arginine treatment (47) and an increased IL-6 production with a decrease TNF-α after treatment with citrulline (42). Asgeirsson et al. showed an opposite result in rats that oral citrulline supplementation impacted the proinflammatory mediators response by decreasing IL-6 in sepsis (48). These inconsistent results may due to different species of rats as well as a different age group of the study subjects. In cell culture study, we used an arginine-enriched medium, and this may also contribute to different consequence.

**Figure 6:**
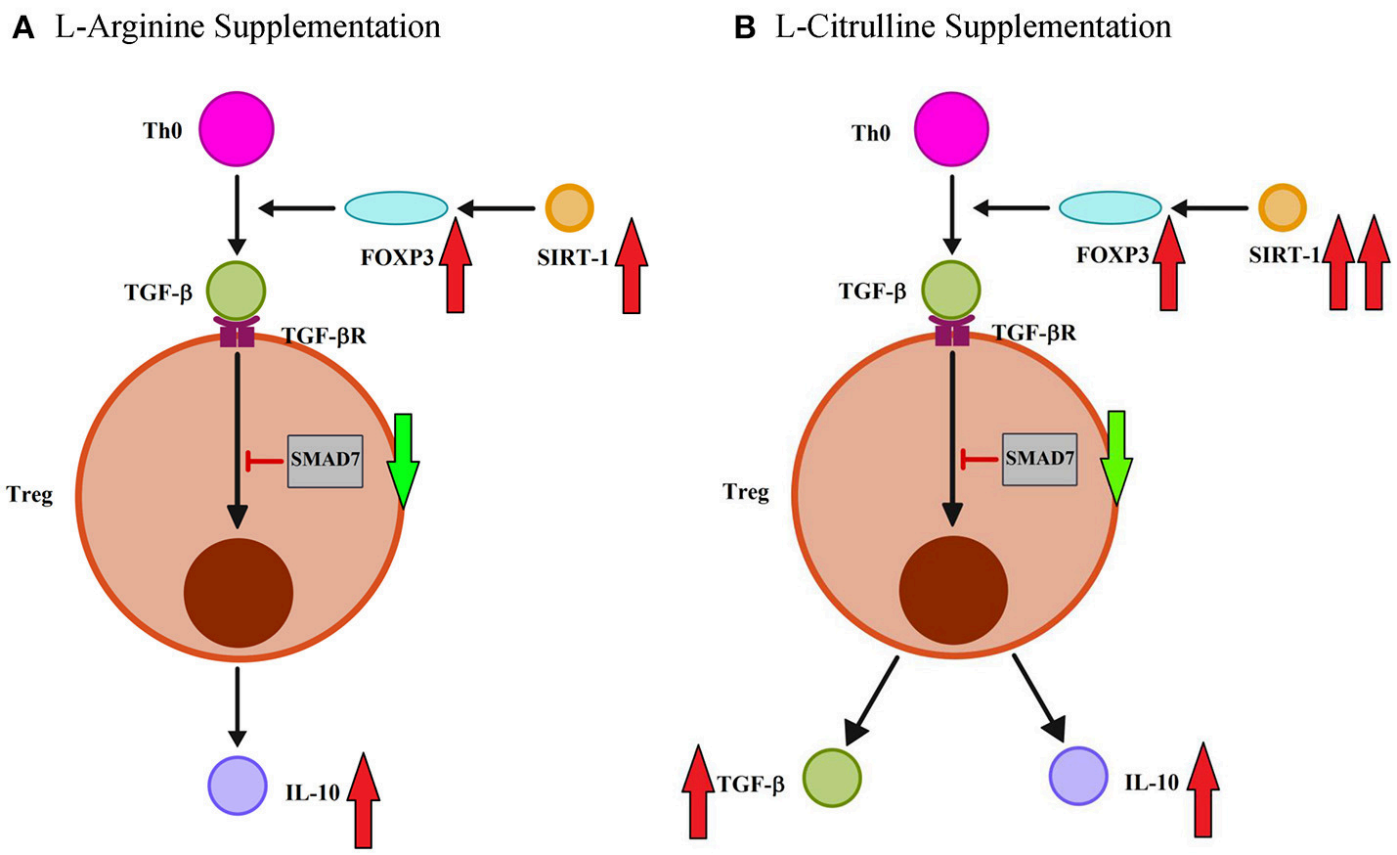
L-arginine and L-citrulline supplementation modulate the Treg function distinctly for infants. **(A)** L-arginine therapy modulate regulatory T-cell (Treg) immune effects by increasing IL-10 rather than TGF-β production. L-arginine treatment also enhance TGF-β signaling by decreasing SMAD7 expression and enhance SIRT-1 abundance. **(B)** In contrast, L-citrulline treatment promote both IL-10 and TGF-β productions. L-citrulline treatment also enhance TGF-β signaling by decreasing SMAD7 expression and enhance FOXP3/SIRT-1 molecule.

When we assessed the effect of amino acid supplementation on the adaptive immune response, we found that postnatal L-arginine treatment enhanced Th1 immune response by increasing IFN-γ production while IL-4 and IL-13, IL-17A were not affected at all. There was no obvious effect on the Th1/Th2 cytokines in the L-Cit group. We then studied the effect of amino acid treatment on Treg and we found that L-arginine supplementation was able to increase the production of IL-10 but not TGF-β1. This finding was consistent with our previous result showing exogenous L-arginine supplementation enhance IL-10 rather than TGF-β production of cord blood CD4+ T-cells (36). While L-citrulline therapy enhanced Treg of infant rats by promoting both IL-10 and TGF-β1 production. Both L-arginine and L-citrulline therapy were also noted to decrease SMAD7 expression, an inhibitor of TGF-β receptor signal pathway, and enhance SIRT-1 abundance. However, FOXP3 expression was only modulated by L-citrulline treatment. Thus, L-arginine and L-citrulline supplementation have different modulatory effect for T-cells function of infants (Figure 6). To our knowledge, this is the first experiment on the modulatory effect of L-citrulline on Treg response in the literature.

More than different effects, it seems that L-citrulline supplementation has a more potent effect than L-arginine supplementation in the modulation of the immune response for newborn. Once L-arginine is orally administered, it is extensively catabolized by arginase in the gut and liver (45, 49). This may limit its bioavailability as a substrate for NOS (45). Previous reports demonstrated that L-citrulline is an potent precursor of L-arginine, thus contributing to sustained L-arginine supply for nitrogen homeostasis (49). L-citrulline supplementation was even observed to increase plasma L-arginine levels in healthy human volunteers more effectively than L-arginine itself in equivalent dose (45). In contrast to L-arginine, previous researches have demonstrated that L-citrulline suppresses arginase activity, acting as a strong allosteric inhibitor (50). Collaborate with more abundant arginase in neonatal leukocytes (18), these could explain the more potent effect of L-citrulline than L-arginine in the modulation of the immune response for newborn.

SMAD molecules involve the signaling pathway of TGF-β has been well documented (28). The receptor-regulated SMADs (R-SMADS, SMAD1,2,3,5,8) are involved in direct signaling from the TGF- receptor (51). Common SMAD (Co-SMAD, SMAD4) cooperate with R-SMADS to form SMAD complex and controls expression of target gene with other transcription factors (52). This signaling pathway negatively controlled by the inhibitor-SMAD (I-SMAD, SMAD6, and 7). SMAD7 is the general antagonist of TGF-β family signaling and exert its inhibitory effects at the receptor level or transcription level (53). Previous studies have shown that SMAD7 knockout mice exhibited an augmented TGF-β induced signaling (54). In our study, we found the expression of SMAD7 mRNA is decreased by both L-arginine and L-citrulline treatment. Thus, both L-arginine and L-citrulline supplementation may promote TGF-β down-stream signal pathway even though only L-citrulline supplementation enhanced the production of TGF-β.

FOXP3 is a major transcription factor for Treg and it activate IL-10 and TGF-β1 production (55). SIRT-1 is a class III histone deacetylase and its immune regulatory role has become more prominent in recent years study. SIRT1 is well known to involve extensively in many physiological as well as pathological conditions such as aging, cancer, neurodegenerative diseases and metabolic processes (56). The regulatory role of SIRT-1 in the immune system has been revealed recently. SIRT-1 was reported to inhibit the differentiation and function of Treg through deacetylating and destabilizing FOXP3, leading to the decrease of TGF-β1 and Th1 promotion (56, 57). To our surprise, we did not observe the expected decrease in SIRT-1, with its reciprocal regulation of FOXP3, after L-arginine and L-citrulline treatment. In contrast, we found a consistent increase for SIRT-1 and Treg response with exogenous L-arginine and L-citrulline supplementation. This is in agreement with another study which also found genetic deletion of SIRT1 in DCs inhibited the generation of T reg cells (58). Another report also revealed that splenic myeloid-derived suppressor cells from SIRT-1 knock-out mice produce lower IL-10 and TGF-β than wild type (59). Thus, the role of SIRT-1 for Treg with L-arginine and L-citrulline therapy need to be clarified in future studies.

NO plays an important role in many physio-pathological conditions in brain, either as a signaling molecule or as a cytotoxic host defense mechanism (60, 61). Adequate NO generation is dependent on proper supply of L-arginine. Under proin flammatory conditions, argininosuccinate synthetase expression is increased in glioma cells and astroglial cultures, a functional role in the recycling of L-citrulline to generate L-arginine for the production of NO has been demonstrated (62). However, when released in excess, iNOS-derived NO can be harmful to the host. In neonatal hypoxia-ischemia model, excessive NO combine with superoxide radicals to produce oxidative stress and result in mitochondrial dysfunction and neuronal toxicity has been demonstrated (63). Thus, the effects of both L-arginine and L-citrulline on neonatal brain and potential neuroinflammatory responses need to be further studied.

Our study has several limitations. First, we administrated the amino acids via intraperitoneal injection rather than oral supplementation. This is because we wanted to study the supplementary effects of indicated amino acids for infant rats while they are still un-weaned. Thus, we do not know whether the immune regulatory effect of indicated amino acid supplementation via a different route will be similar or not. Besides, we did not use arginine free culture medium for cell culture and thus might mask or influence the possible effects of the supplementation of arginine or citrulline on the immune response. However, we have provided evidences showing the sustained immune regulatory effects of L-arginine and L-citrulline supplementations even when splenocytes are later culture in amino acid enriched medium.

In conclusion, we have shown that with the addition of L-arginine, Th1 immune response is activated through increase of IFN-γ production. While supplement with L-arginine and L-citrulline to infantile rats have distinct Treg immune modulatory effects. The possible mechanism of modulation in Treg is through FOXP3, SMAD7 and SIRT-1 regulation. Exogenous supplementation of indicated amino acids has the potential to be a strategy for infants in immune dysregulated conditions.

## Author Contributions

H-RY, Y-CL, and T-YL contributed to designed the work. H-RY, Y-TS, C-HC, and Y-CL: contributed to data acquisition. H-RY, Y-CL, Y-TS, and C-HC performed data analysis and interpretation. H-RY, Y-CL, C-MT, and C-HC drafted the manuscript. H-RY, Y-CL, C-MT, and C-HC finalized the article. All authors have read and approved the final manuscript and agreed to be accountable for all aspects of the work.

### Conflict of Interest Statement

The authors declare that the research was conducted in the absence of any commercial or financial relationships that could be construed as a potential conflict of interest.
